# Surface-Oxidised Carbon Nanofibre-Based Nanofluids: Structural, Morphological, Stability and Thermal Properties

**DOI:** 10.3390/nano12213922

**Published:** 2022-11-07

**Authors:** Norshafiqah Mohd Saidi, Norli Abdullah, Mohd Nurazzi Norizan, Nurjahirah Janudin, Noor Azilah Mohd Kasim, Mohd Junaedy Osman, Imran Syakir Mohamad, Mohd Afzanizam Mohd Rosli

**Affiliations:** 1 Centre for Defence Foundation Studies, Universiti Pertahanan Nasional Malaysia, Kem Sungai Besi, Kuala Lumpur 57000, Malaysia; 2 Bioresource Technology Division, School of Industrial Technology, Universiti Sains Malaysia, Penang 11800, Malaysia; 3 Green Biopolymer, Coatings & Packaging Cluster, School of Industrial Technology, Universiti Sains Malaysia, Penang 11800, Malaysia; 4 Research Centre for Chemical Defence, Universiti Pertahanan Nasional Malaysia, Kem Perdana Sungai Besi, Kuala Lumpur 57000, Malaysia; 5 Faculty of Mechanical Engineering, Universiti Teknikal Malaysia Melaka, Hang Tuah Jaya, Durian Tunggal, Melaka 76100, Malaysia

**Keywords:** carbon nanofibre, CNF, nanofluids, polyvinylpyrrolidone, thermal conductivity, surface oxygen functional group, sedimentation

## Abstract

The reputation of nanofluids as a convenient heat transfer media has grown in recent years. The synthesis of nanofluids is often challenging, particularly carbon-based nanofluids, due to the rapid agglomeration of the nanoparticles and the instability of the nanofluids. In this regard, surface modification and surfactant addition are potential approaches to improve the physical and thermal properties of carbon-based nanofluids that have been studied and the structural, morphological, and thermal characteristics of surface-oxidised carbon nanofibre (CNF)-based nanofluids has been characterised. Commercial CNF was first subjected to three different acid treatments to introduce surface oxygen functional groups on the CNF surface. Following the physical and thermal characterisation of the three surface-oxidised CNFs (CNF-MA, CNF-MB, and CNF-MC), including Raman spectroscopy, Fourier transform infrared (FTIR), thermogravimetric analysis (TGA), and field emission scanning electron microscopy (FESEM), the CNF-MB was selected as the best method to synthesise the surface-oxidised CNF-based nanofluid. A total of 40 mL of ultrapure water was used as a pure base fluid and mixed with the surface-oxidised CNF at a concentration range of 0.1–1.0 wt.%, with a fixed of 10 wt.% amount of polyvinylpyrrolidone (PVP). The thermal conductivity of CNF-based nanofluid was then characterised at different temperatures (6, 25, and 40 °C). Based on the results, surface oxidation via Method B significantly affected the extent of surface defects and effectively enhanced the group functionality on the CNF surface. Aside from the partially defective and rough surface of CNF-MB surfaces from the FESEM analysis, the presence of surface oxygen functional groups on the CNF wall was confirmed via the Raman analysis, TGA curve, and FTIR analysis. The visual sedimentation observation also showed that the surface-oxidised CNF particles remained dispersed in the nanofluid due to the weakened van der Waals interaction. The dispersion of CNF particles was improved by the presence of PVP, which further stabilised the CNF-based nanofluids. Ultimately, the thermal conductivity of the surface-oxidised CNF-based nanofluid with PVP was significantly improved with the highest enhancement percentage of 18.50, 16.84, and 19.83% at 6, 25, and 40 °C, respectively, at an optimum CNF concentration of 0.7 wt.%.

## 1. Introduction

Heating and cooling fluids are employed in numerous industrial settings, including energy supply and generation, electronics, chemical engineering, solar thermal collectors, and automotive. Recently, heat transfer media have drawn the interest of researchers as it is able to transfer heat from one liquid component to another in any process, which makes them a vital component to prevent overheating and severe system breakdown of electronic or automobile applications. In view of this, extensive research has been focused on nanofluids, which are liquid suspensions of nanometre-sized solid particles, including metal oxides, such as titanium dioxide (TiO_2_) and zinc oxide (ZnO), metals, and carbon-related materials, such as carbon nanofibre (CNF), carbon nanotube (CNT), and graphene, due to their potential application as heat transfer fluids [1,2]. Comparatively, nanofluids exhibit better convective heat transfer capabilities and greater heat conductivities than that of conventional fluids (such as water and ethylene glycol) and at the same time, are able to mitigate the issues of sedimentation, erosion, and clogging related to the suspensions of larger than milli- or micrometre-sized particles [3]. Nevertheless, nanometre-sized liquid suspensions exhibit several downsides, including the potential contamination in the heat transfer media and the agglomeration of nanoparticles that would obstruct the device’s channel, consequently minimising the efficiency of incorporation of nanoparticles in the base fluid for heat transfer applications. Since the stability of nanoparticles in any given fluid is directly associated with the electrokinetic characteristics of the solid–liquid mixture, the synthesis of a stable nanofluid should consider the proper control of pH solution, surfactant addition, and surface modification of the suspended particles. Hence, the rapid sedimentation should be addressed and eliminated in the early preparation stage and synthesis of nanofluids in order to examine the long-term suspension stability of nanofluids [4,5,6].

CNFs in aqueous media have the potential to be a good heat transfer media in heat exchangers, cooling devices, and solar collectors due to their unique mechanical and thermal properties [7,8]. However, the inability of CNFs to disperse homogeneously in most solvents limits practical uses significantly. Furthermore, due to their very nonpolar surface, CNFs are unable to interact and have a good bonding with a variety of matrix materials. Adding a new functional group or other material, such as metal or polymer, converts hydrophobic CNFs to hydrophilic CNFs, considerably enhancing dispersibility and stability in aqueous media [9]. This need modification involves two types of covalent and non-covalent functionalization on the CNFs sidewall. Their high aspect ratios cause CNF to entangle and form bundles. CNF have high van der Waals interaction energy of the nanofibre contact which causes a strong bundling of CNF. This high interaction force makes the dispersion stability a challenging task. To overcome this problem, two different approaches are currently being used to disperse carbon nanotubes, i.e., mechanical (or physical) methods and chemical methods. By using mechanical methods such as ultrasonication and high-shear mixing, nanotubes are separated from each other. However, these methods can also fragment long nanofibres, causing their aspect ratio to decrease during processing, and these are time-consuming and inefficient methods [10,11,12].

Chemical methods use surfactants or chemical moieties to change the surface energy of the nanotubes, improving their wetting or adhesion characteristics and improving their dispersion stability in solvent. Covalent functionalisations include the bonding of different chemical functional groups on the sidewalls of CNF. However, aggressive chemical functionalization causes an increase in the defects on the sidewalls. This can alter the electrical and mechanical properties of CNF. Furthermore, covalent functionalization changes the electric properties by altering their binding energy, but the covalent functionalization is more stable and referred for heat transfer media. Some examples of covalent functionalization are surface oxidation, esterification, amidation, and fluorination [6,13] that insert into the sidewall of CNFs. Usually, the esterification and amidation functionalization proceed after CNF undergoes acid treatment. In contrast, the non-covalent approach involves adsorption of chemical moieties on the surface of CNF by involving weak interaction bonding such as π-π stacking or through Coulombic attraction in case of charged chemical moieties, hydrogen bond, hydrophilic or hydrophobic force, and electrostatic force and does not alter the electrical properties of CNF but this functionalization is unstable [14].

An alternative approach to enhance the heat transfer capacity of nanofluids is through the introduction of surface oxygen functional groups via acid treatment [15]. This method employs the insertion of oxygen functional groups, for example, carbonyl group (C=O), carboxyl group (-COOH), and hydroxyl group (-OH), on the sidewall of carbon particles to promote defects on the nanofluid surface [16]. Furthermore, the oxygen functional groups would boost the interaction between the water molecules and carbon particles, causing the hydrophobic carbon particles to be more hydrophilic and greatly improving their dispersibility. Thus, surface modification using oxygen functional groups offers an effective optional approach to improve the thermal conductivity of heat transfer media for various applications.

Realising the potential of such approaches, this study was performed to analyse the structural, morphological, and thermal properties of surface-oxidised CNF-based nanofluids. Primarily, surface modification of commercial CNF was carried out using three different acid treatments (Methods A, B, and C) to introduce surface oxidative functional groups on the surface of the CNF. The impact of surface oxidative functional groups on the structural, morphological, and thermal characteristics of the surface-oxidised CNF was then characterised. The method that produced the optimal characteristics of the surface-oxidised CNF was then applied to prepare the CNF-based nanofluids with and without the presence of the PVP as the surfactant. Finally, the performance of the synthesised surface-oxidised CNF-based nanofluid was evaluated through the stability and dispersion analysis as well as the thermal conductivity tests.

## 2. Experimental Procedures

### 2.1. Materials

Commercial carbon nanofibres (CNF)-HHT24 was acquired from Pyrograf Product, Inc. The HHT24 code denotes the high heat treatment applied during the synthesis of the CNF. Meanwhile, nitric acid (HNO_3_, 65%) and concentrated sulphuric acid (H_2_SO_4_, 98%) were procured from Merck kGaA, Darmstadt, Germany for the surface oxidation of CNF. In addition, polyvinylpyrrolidone (PVP) was acquired from R&M Chemicals, Essex, the United Kingdom as the surfactant for the nanofluid synthesis. All chemicals used in the present study were of analytical reagent (AR) grades and applied directly without any purification. The detailed properties of the CNF-HHT24, H_2_SO_4_ and HNO_3_, and PVP are presented in Table 1, Table 2 and Table 3, respectively.

### 2.2. Surface Oxidation Treatments of CNF

**Figure 1:**
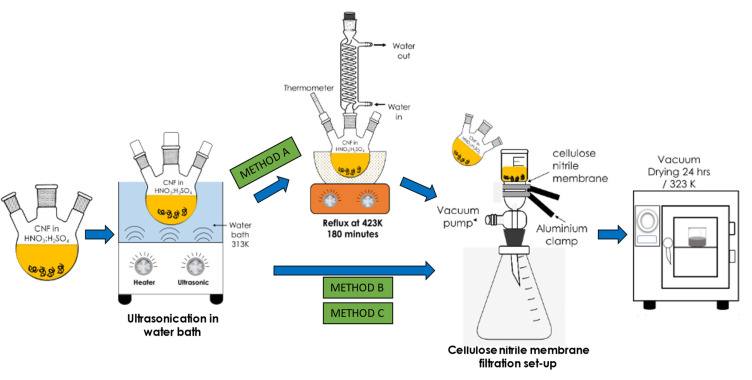
Schematic representation of the three varying surface oxidation treatments of the commercial CNF.

Three preliminary acid treatments, namely Method A, Method B, and Method C, were performed to modify the commercial CNF. In Method A, approximately 2 g of commercial CNF was mixed with 98% concentrated H_2_SO_4_ and 65% HNO_3_ (acid ratio of 3:1 (*v/v*)) in three neck round bottom flasks. The flasks were then transferred in an ultrasonicate water bath, FB 15057 (Fisher Scientific, Waltham, MA, USA) for 30 min at a frequency of 50/60 Hz and temperature of 313 K to disentangle the CNF aggregates/agglomerates and to provide anchoring oxygen functional groups on the CNF surface. Subsequently, the mixture was subjected to the reflux treatment for 180 min at 423 K before the obtained modified CNF powder was rinsed with deionised water a few times and filtered using a cellulose nitrile membrane filter (pore size of 0.43 µm) until the pH reached 7.0. Finally, the CNF powder was dried for 24 h in a vacuum oven at 323 K. Meanwhile, both Methods B and C follow the same steps as in Method A except that the reflux treatment was excluded. In addition, the ultrasonication duration for Method B and Method C was fixed at 2 h and 6 h, respectively, at 343 K. The overall steps for the surface oxidation treatments of CNF are illustrated in the schematic diagram in Figure 1.

### 2.3. Characterisation of Surface-Oxidised CNF

The structural evaluation of the surface-oxidised CNF from all three methods was characterised using a Perkin Elmer Frontier Fourier Transform Infrared (FTIR) spectrometer (BX FTIR (Perkin Elmer, Beaconsfield, IA, USA)) with the number of scan and scan range of 16 and 500–4500 cm^−1^, respectively. Prior to the analysis, the sample was prepared by mixing a small concentration of the CNF samples with potassium bromide (KBr) powder (sample-to-KBr ratio of 1:9) and compressed into a pellet. In addition, the structural characterisation of the surface-oxidised CNF was determined using a Renishaw inVia Reflex Confocal Micro Raman System (Renishaw plc, Wotton-under-Edge, UK) following the method by Nurazzi et al. (2021) [17]. For the sample excitation, the HPNIR laser was programmed at a wavelength, grating, and magnification of 787 nm, 1200 mm^−1^_,_ and 100× objective lens, respectively. Meanwhile, a JEOL 7600F Field Emission Scanning Electron Microscopy (FESEM) Gemini 500 (Carl Zeiss AG, Jena, Germany) was employed to examine the surface morphology and diameter distribution of the commercial CNF before and after the surface oxidation treatment. The samples were first platinum (Pt)-coated for 30 s to prevent charging. The diameter of the CNF nanoparticles was then measured using the ImageJ software with 50 points for each CNF sample. Furthermore, the Thermogravimetric Analysis (TGA) was carried out to analyse the thermal stability of the surface-oxidised CNF using a TGA-DSC HT 3 analyser (Mettler Toledo, Selangor, Malaysia) at a temperature range and heating rate of 35–1000 °C and 5 °C/min, respectively, under a fixed nitrogen gas flow.

### 2.4. Preparation of Surface-Oxidised CNF-Based Nanofluid

The preparation method of the surface-oxidised CNF-based nanofluids was carried out based on the best surface oxidation treatment between CNF-MA, CNF-MB, and CNF-MC with and without the addition of PVP as the surfactant. Initially, 40 mL of ultrapure water was collected from a Milli-Q Direct Water Purification System into a glass container, which serves as the base fluid for the nanofluid synthesis. In addition, the developed surface-oxidised CNF was added into the water at a range of 0.1–1.0 weight concentration (wt.%) with an interval of 0.1. Meanwhile, a 10 wt.% PVP was prepared prior to the nanofluid synthesis. The non-ionic surfactant was then added to the CNF suspension at a range of 0.01–0.10 wt.% to reduce the surface tension within the nanofluid.

**Figure 2:**
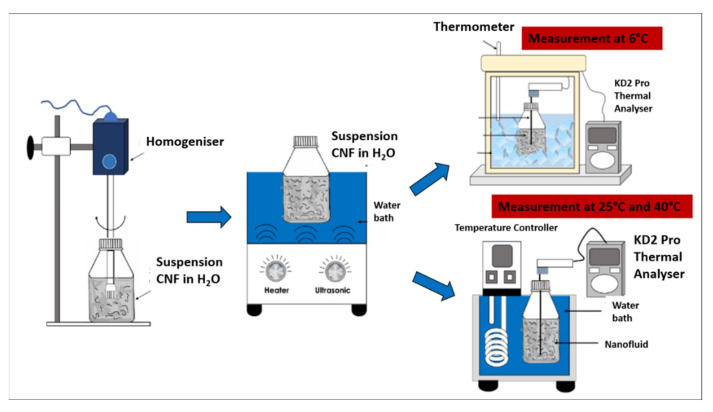
Process flow diagram for the preparation and evaluation of the surface-oxidised CNF-based nanofluids.

The CNF nanoparticles were physically dispersed in the water-based nanofluid via the two-step homogenisation and sonication process using a D1000 Handheld Homogeniser (Benchmark Scientific Inc., Sayreville, USA) for 5 min at a rotation speed of 10,000 rpm, followed by an FB 15057 Ultrasonic Water Bath (Fisher Scientific, Massachusetts, USA) for 30 min at 25 °C and 60 Hz with an output power generation of 240 W. The two-step process was crucial to ensure that the carbon particles in the water-based nanofluids are uniformly dispersed and prevent the agglomeration of the nanofluids through the breakdown of the van der Waals interaction between the particles. Figure 2 illustrates the process flow diagram of the preparation and evaluation of the surface-oxidised CNF-based nanofluids.

### 2.5. Physical and Thermal Characterisation of Surface-Oxidised CNF-Based Nanofluid

The clustering and stability of the surface-oxidised CNF-based nanofluids with and without the presence of PVP were analysed via the sedimentation photograph capturing method. The sedimentation observation of each nanofluid sample was photographed using a smartphone camera after 0.5 h and 100 h of the homogenisation and sonication process [18]. CNF is naturally hydrophobic and generally exists in bundles. Concurrently, CNF exhibits an extremely high aspect ratio, which facilitates it agglomerating into various shapes and sizes. In other words, rapid agglomeration and sedimentation take place when CNFs are dispersed in a base fluid, making it challenging to maintain their stability, especially for suspensions containing carbonaceous particles. Thus, a nanofluid with no particle sedimentation is considered stable. Hence, a pure base fluid was prepared as a constant to compare the physical and thermal properties of the surface-oxidised CNF-based nanofluids.

**Figure 3:**
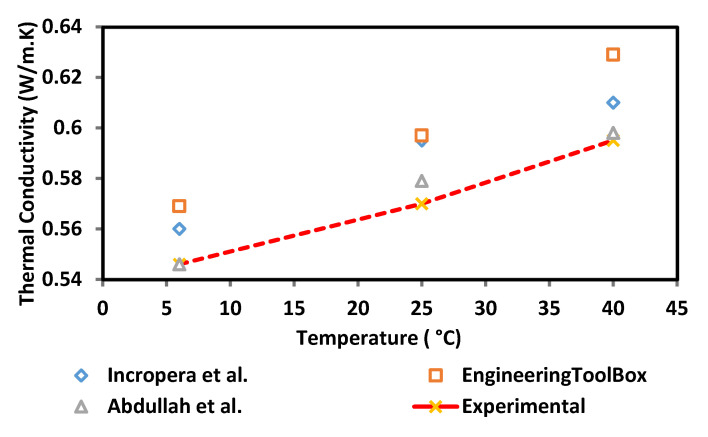
Comparison of thermal conductivity validation between experimental and reference values.

The thermal conductivity of the surface-oxidised CNF-based nanofluid was evaluated at three varying temperatures of 6, 25, and 40 °C using a KD2 Pro Analyser (Decagon, Pullman, WA, USA) equipped with a water-based fluid, KS-1 (60 mm long × 1.3 mm diameter) at a 5% specific accuracy. The KD2 Pro Analyser is calibrated with a standard glycerine solution at room temperature each time before measurement. The nanofluid sample was first placed in a vessel and the needle sensor was inserted into the sample. Both the sample and needle sensor were maintained for 30 min at the required temperature. Once the temperature was stable, the thermal conductivity was recorded three times at 15 min intervals between each measurement. The calibration of KD2 Pro Analyser was carried out using a standard glycerine solution at room temperature prior to every evaluation and the experiment was performed in a secluded environment to minimise the fluctuation error. The preliminary experimental study was performed to establish and accuracy of our measurement data in comparison with reference values from Bergman et al. [19], Abdullah et al. [20] and EngineeringToolBox [21]. Figure 3 shows the comparison of thermal conductivity between experimental and reference values of 100% deionised water at three different temperatures (6, 25, and 40 °C). There is a good agreement between the experimental and reference values. From this result, it shows that the instrument (KD2 Pro Analyser) is reliable and accurate especially for high viscosity liquid. The experimental values (100% ultrapure water) used as a standard or datum to calculate the thermal conductivity of enhancement of CNF based nanofluids.

## 3. Results and Discussion

### 3.1. FTIR Spectra Analysis

**Figure 4:**
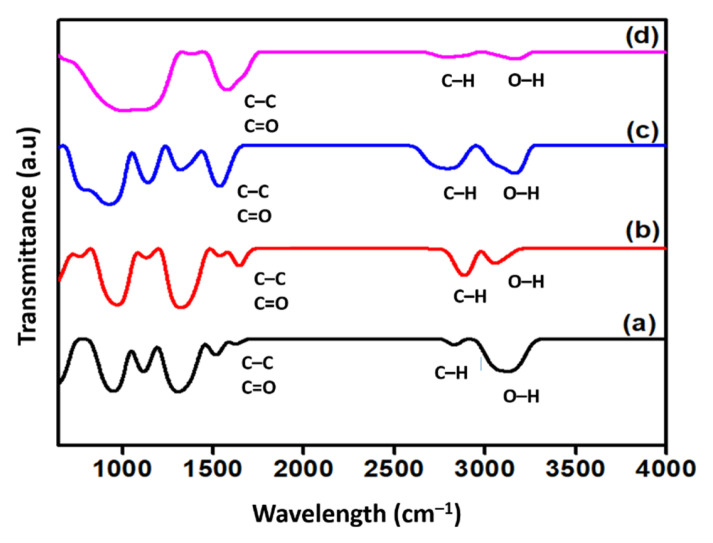
FTIR spectra of (**a**) commercial CNF and the surface-oxidised (**b**) CNF-MA; (**c**) CNF-MB; and (**d**) CNF-MC.

Figure 4 shows the qualitative change of the FTIR spectra of the surface-oxidised CNF. Generally, all spectra exhibited two distinct peaks around 1660 and 3100 cm^−1^ that corresponded to the C=O stretching vibrations and -OH groups, respectively. The detection of the -OH groups on the surface of the commercial CNF was assumed either to be due to the ambient atmospheric moisture or the effect of oxidation during the purification of CNF [22]. Additionally, the peak at approximately 1580 cm^−1^ in all spectra was associated with the C-C stretching vibration of the polyaromatic C=C bond [16]. In addition to that, two specific bands were detected at approximately 2900 and 2800 cm^−1^ in all FTIR spectra, which correspond to the C-H stretching vibration of the carboxyl group, which normally indicates a defect in the CNF rings [23]. Moreover, the increased intensity of the double bands at 2800 and 2900 cm^−1^ of the surface-oxidised CNFs, particularly in CNF-MA and CNF-MB, was due to the covalently attached hydroxyl and carboxyl groups on the CNFs during the surface modification treatment. Eventually, this finding verified the occurrence of the oxidation process on the CNF surface.

### 3.2. Raman Spectroscopy Analysis

**Figure 5:**
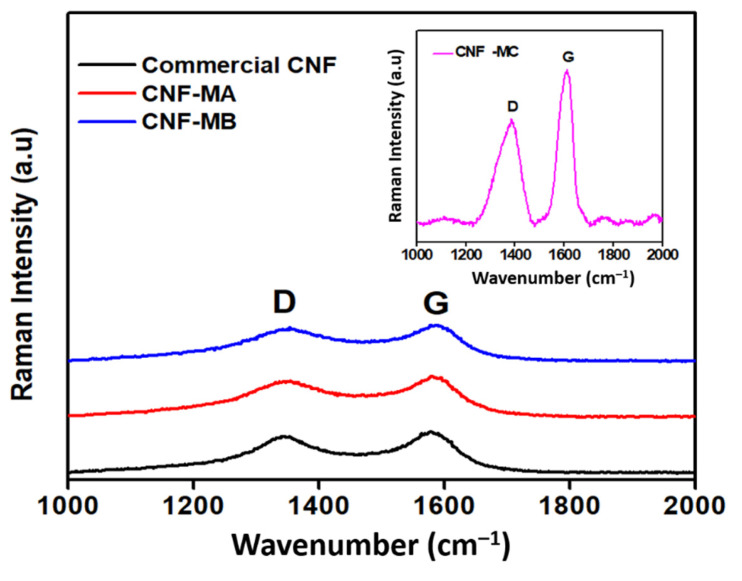
Raman spectra of the commercial CNF, CNF-MA, and CNF-MB samples; insert figure is the Raman spectra of CNF-MC.

Figure 5 shows the Raman spectroscopy analysis of the commercial and surface-oxidised CNF samples. The Raman spectra determined two notable peaks that correspond to the CNF properties in the peak range of 1250–1450 cm^−1^ (D-band) was designated to the corresponds to the presence of amorphous carbon and defects in the structure of carbon atoms hybridised as type sp^2^, while the second peak range of 1500–1605 cm^−1^ (G-band) was attributed to the two-dimensional (2D) hexagonal lattice of the sp^2^-bonded carbon atoms, signifying stretching of the C–C bond in graphitic materials and is common to all sp^2^ carbon systems. Furthermore, the peak intensity ratio (R) of the D-band to the G-band (I_D_/I_G_) in Table 4 correlates to the number of deformed sites on the CNF surface. Apart from that, two main peaks were detected in all four CNF samples, namely the D-band and the G-band at 1339 cm^−1^ and 1585 cm^−1^, respectively. The latter peak marginally shifted by 5–6 cm^−1^ indicates an increase in I_D_/I_G_ after an oxidative treatment of CNF, therefore, indicates an increment in the number of defect sites on the nanofibers compared to the commercial CNF as a result of the presence of the oxygen functional group on the CNF surface. Therefore, it is suggested that the presence of the D peak results from structural defects in the CNF due to the acid surface treatment. Peak G (at 1585 cm^−1^) arises from the stretching of the C–C bond in graphitic materials and is common to all sp^2^ carbon systems [24]. Based on the I_D_/I_G_ ratio of CNF-MA and CNF-MB samples, it was deduced that the introduction of surface oxygen functional groups, such as the carboxylic acid, carbonyl, hydroxyl, or ether groups. Among others, from the acid treatment induced only minimal defect sites on the CNF wall [25]. In comparison, the Raman peak was significantly intense in the CNF-MC sample with an I_D_/I_G_ ratio of 1.06 with longer ultrasonication process (6 h). Increasing the oxidation time through the ultrasonication process longer than 2 h would create more reactive sites on the fibre surface; however, at the same time it would damage more of the surface area. Therefore, longer times are not appropriate because they result in a deterioration of the fibres, resulting in a loss of their surface structure under the FESEM image and thermal stability characteristic.

### 3.3. FESEM Analysis

**Figure 6:**
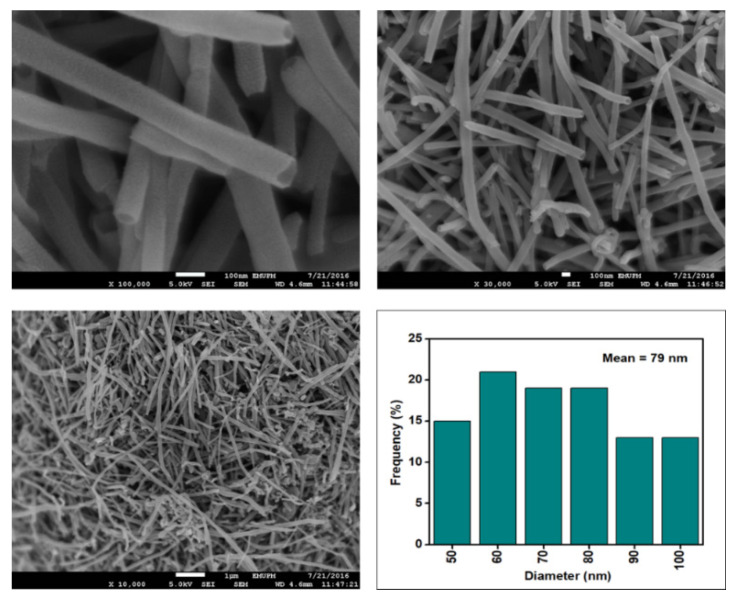
FESEM imaging of the commercial CNF at 100, 30, and 10 resolutions and their corresponding diameter distribution histogram (mean diameter = 79 nm).

**Figure 7:**
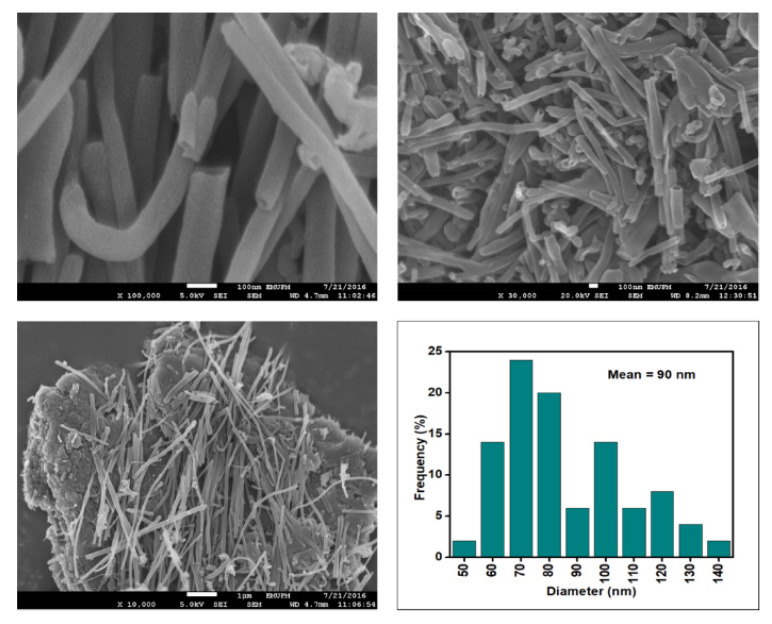
FESEM images of the CNF-MA at 100, 30, and 10 resolutions and their corresponding diameter distribution histogram (mean diameter = 90 nm).

**Figure 8:**
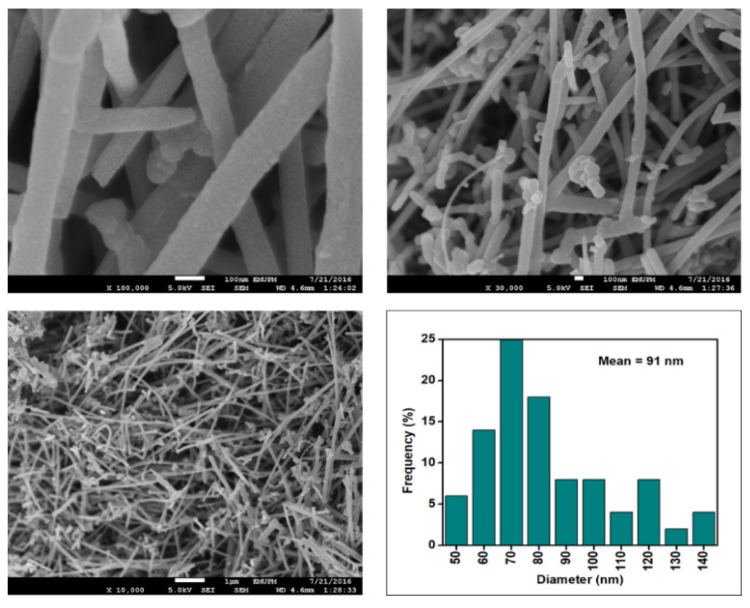
FESEM images of the CNF-MB at 100, 30, and 10 resolutions and their corresponding diameter distribution histogram (mean diameter = 91 nm).

**Figure 9:**
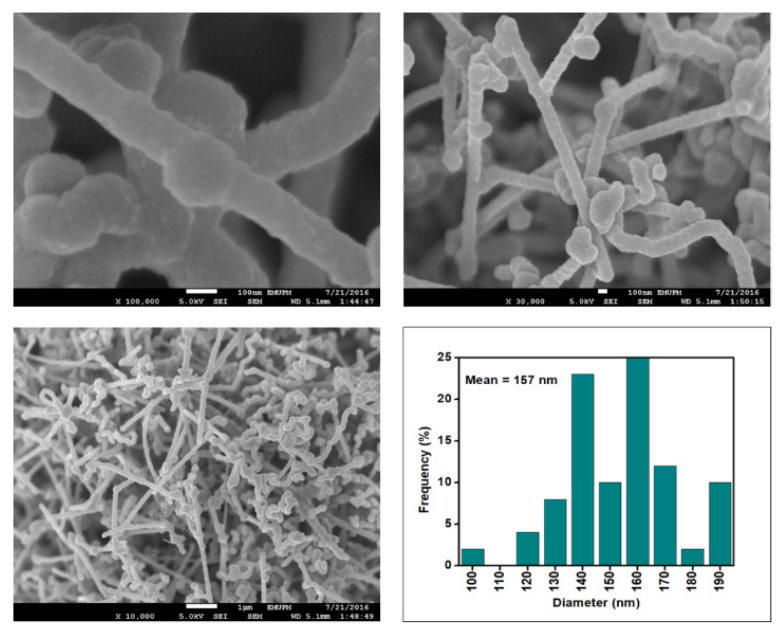
FESEM images of the CNF-MC at 100, 30 and 10 resolutions and their corresponding diameter distribution histogram (mean diameter = 157 nm).

Figure 6, Figure 7, Figure 8 and Figure 9 depict the surface morphology of the commercial and surface-oxidised CNF from the FESEM analysis, which corresponds to the diameter distribution histograms at varying resolutions. The surface of the commercial CNF was relatively smooth with a diameter distribution range of 50–100 nm and a mean diameter of 78 nm. Following the surface oxidation treatment using Method A, the molecular organisation of the nanoparticles was more arranged and denser, while the diameter distribution slightly increased with a mean diameter of 90 nm. Comparatively, the surface morphology was drastically altered following Method B. As shown in Figure 6, certain parts of the CNF-MB surfaces were partially defective and rough. This is consistent with the impact of acid mixture between H_2_SO_4_ and HNO_3_, which is typically applied to split the highly tangled long fibre of nanotubes [26]. However, the CNF-MC showed a more disrupted and rough surface structure on the side wall of the CNF. Additionally, the mean diameter of the CNF-MC increased to 157 nm with a larger diameter distribution range of 100–190 nm. It was assumed that the prolonged exposure of the CNF to high temperature influenced the surface wall alteration and induced the expanding thickness of the CNF wall. The result corresponded to the greater defect sites on the CNF-MC wall and the highly intense peak from the Raman analysis.

A representative elemental analysis of the commercial and oxidised CNFs was performed using energy dispersive X-Ray (EDX) on several spots. The main elements and concentrations found are listed in Table 5. The largest element in all the CNFs samples is carbon followed by oxygen content. A small content of Al is also detected in the commercial CNFs, and oxidised CNFs.

The oxygen concentration increases with treatment time, as indicated in Table 5 The oxygen concentration of commercial CNFs is 7.44 wt.%, increases to 8.29 wt.% and 9.69 wt.%, whereas, when CNFs are treated with a combination of HNO3/M H2SO4 for a longer period of time, the oxygen content nearly doubles (19.52 wt.%). This suggests that the surface oxygen functional group (SOFG) concentration of acidic and basic groups increased with oxidation period. As a result, when the sonication treatment lasted at least 6 h, the SOFG groups were extensively introduced onto the nanofibres’ surface.

### 3.4. TGA Results

**Figure 10:**
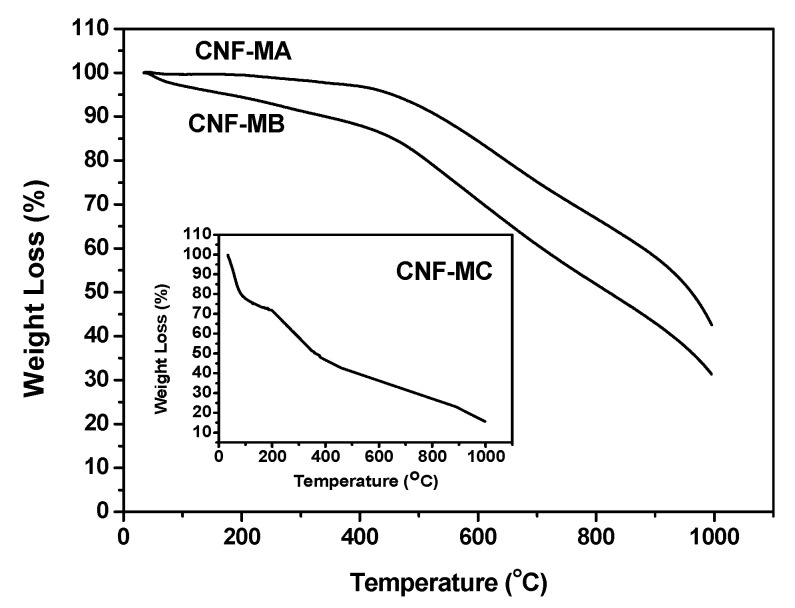
TGA curves representing the weight loss of the CNF-MA (>400 °C), CNF-MB (>450 °C), and CNF-MC (>180 °C).

Figure 10 presents the TGA curves of the surface-oxidised CNFs samples. In general, the weight loss process can be divided into four stages. The first occurs at below 150 °C, which is attributed to the loss of moisture content. The second weight loss occurs from 150 to 400 °C and is ascribed to the decomposition of oxygen functional group presented on the CNFs walls. The third stage in the range between 400 and 500 °C is due to elimination OH and thermal oxidation of the remaining amorphous carbon happened above 500 °C [27].

The CNF-MC recorded the highest weight loss with four different stages compared to that of CNF-MA at 400 °C and CNF-MB at 450 °C. The weight loss of CNF-MB and CNF-MA within the 120 to 450 °C temperature range was due to the hydroxyl group removal and the decarboxylation process [28], while the weight loss of the CNF-MC samples within the 35 to 120 °C range was contributed by the water molecule evaporation from the CNF surface. Interestingly, the early decomposition of the CNF-MC sample was associated with the more damaged and irregular surface structure on the side wall of the CNF which is in good agreement with our FESEM observation. Disordered or amorphous carbons tend to be oxidised at above 500 °C because of their lower activation energies for oxidation or due to the presence of a large number of active sites.

### 3.5. Dispersion and Stability Analysis of the Surface-Oxidised CNF-Based Nanofluids

**Figure 11:**
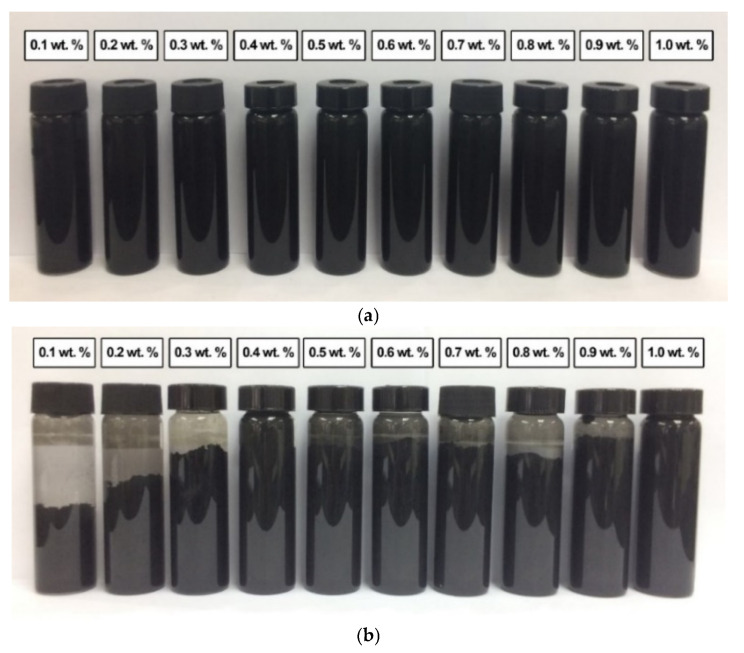
The sedimentation of various commercial CNF-based nanofluids without PVP after (**a**) 0.5 h and (**b**) 100 h of the homogenisation and sonication process.

Figure 11a shows the absence of sedimentation in all commercial CNF-based nanofluids without PVP after 0.5 h of the homogenisation and sonication process. This was due to the slow sedimentation of smaller-sized nanoparticles of the commercial CNF. However, the sedimentation was visible after 100 h at a CNF concentration of 0.1, 0.2, 0.3, 0.8, and 0.9 wt.%, as shown in Figure 11b. Known as mixed sedimentation, this phenomenon occurs when larger agglomerates settled down faster compared to smaller ones [29]. The findings could also be associated with the higher attractive van der Waals forces especially occurred between the carbon-based nanoparticles [30]. In contrast, the sedimentation was unclear at 0.5, 0.6, and 0.7 wt.%, while the 0.4 and 1.0 wt.% remained clear without sedimentation after 100 h. The observation could be attributed to the presence of hydroxyl bonds in the commercial CNFs, as evidenced by the FTIR spectra. Consequently, the hydroxyl groups formed hydrogen bonds with water molecules and induced the hydrophilic properties of some CNF particles.

**Figure 12:**
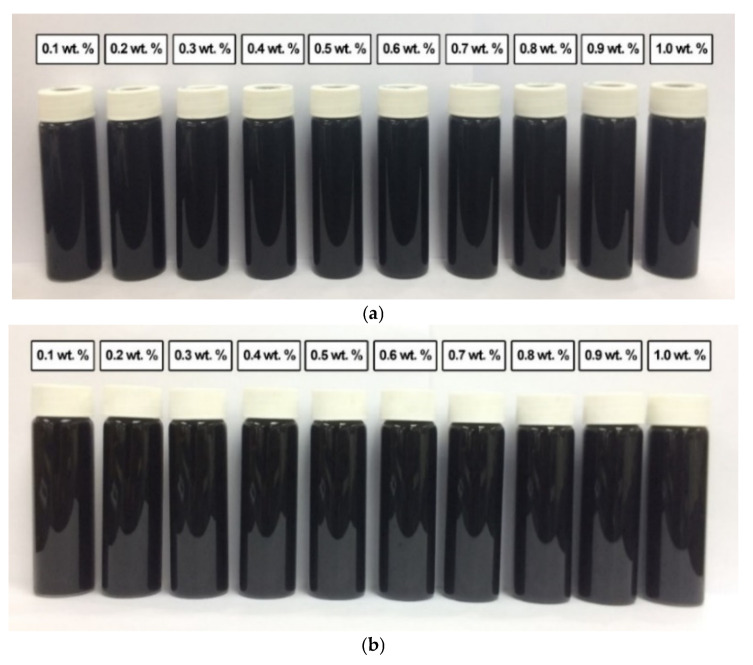
The various surface-oxidised CNF-based nanofluids without PVP after (**a**) 0.5 h and (**b**) 100 h of the homogenisation and sonication process.

In comparison, Figure 12 shows the absence of sedimentation in all surface-oxidised CNF-based nanofluids without PVP after 0.5 h and 100 h of the homogenisation and sonication process. Therefore, all samples were considered stable through the introduction of surface oxygen functional groups (including -COOH and -OH) on the CNF surface, which reduced the van der Waals interaction among the nanoparticles and facilitated their dispersion in the nanofluids compared to that of the commercial CNFs. Apart from the applied sonication process during the surface oxidation treatment, the dispersion process contributed to the energetic effect that allowed the CNFs bundles to remain separate.

**Figure 13:**
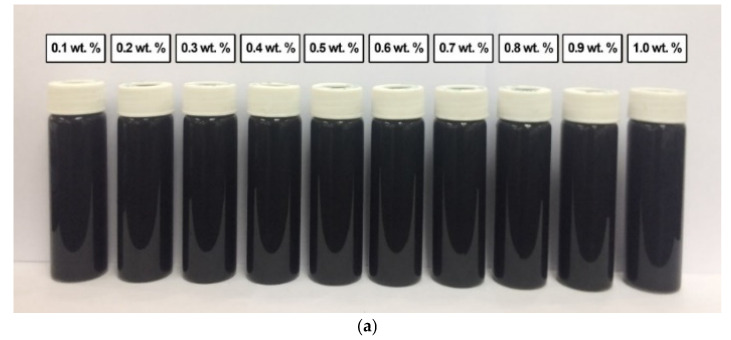

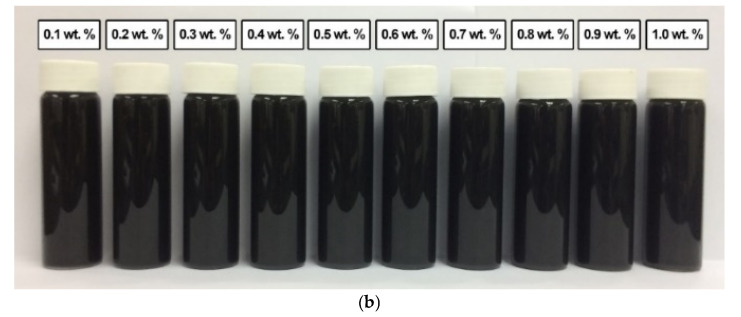
The various surface-oxidised CNF-based nanofluids with the addition of PVP after (**a**) 0.5 h and (**b**) 100 h of the homogenisation and sonication process.

A similar observation was recorded for the surface-oxidised CNF-based nanofluids with the addition of PVP after 0.5 h and 100 h of the homogenisation and sonication process. As shown in Figure 13, the absence of sedimentation after 100 h showed that the presence of surface oxygen functional groups in the CNF-based nanofluids induced the well-dispersion of CNF particles. Furthermore, the sonication process during the surface oxidation treatment contributed to the energetic effect that assisted the CNFs bundles to remain separate. The presence of PVP also further improved the dispersion and stability of the CNF-based nanofluids.

As mentioned earlier, numerous key difficulties impede the synthesis of effective nanofluids, including the agglomeration of nanoparticles and the fast sedimentation rate of nanoparticles in the fluids, where the sedimentation could obstruct the flow channels and severely impact the effective performance of the nanofluid. Among the factors that affect the dispersion and stability of carbon-based nanofluids include the hydrophobic properties of nanocarbons, surfactant addition, modification on the nanocarbon surface, and homogenisation and ultrasonication procedures. For this study, the detection of sedimentation after 100 h in all commercial CNF-based nanofluid samples without PVP was due to the hydrophobicity of the CNF against nanofluid that allows it to form agglomerates. In contrast, the surface-oxidised CNF-based nanofluids without PVP recorded no visible sedimentation after 100 h. Thus, the results proved that the surface oxidation treatment of the CNF prevented the CNFs from agglomerating and improved the overall stability of the nanofluids.

Following the acid treatment, the introduction of the carboxyl group generated negative charges on the surface of the CNF. Subsequently, the increased surface charges led to a decrease in the strong self-interaction of the carbon nanotubes due to the van der Waals attraction. In addition, the presence of PVP as the surfactant enhanced the stability of the nanofluids and impeded the particle sedimentation in water even without the acid treatment process. Overall, the commercial CNF-based nanofluids with PVP were more stable compared to the commercial CNF-based nanofluids without PVP. Although the commercial CNF-based nanofluid with a concentration of 0.1 wt.% showed a minimal formation of sedimentation after 100 h, the sample was considered stable.

### 3.6. Thermal Conductivity of the Commercial CNF-Based Nanofluids without PVP

**Figure 14:**
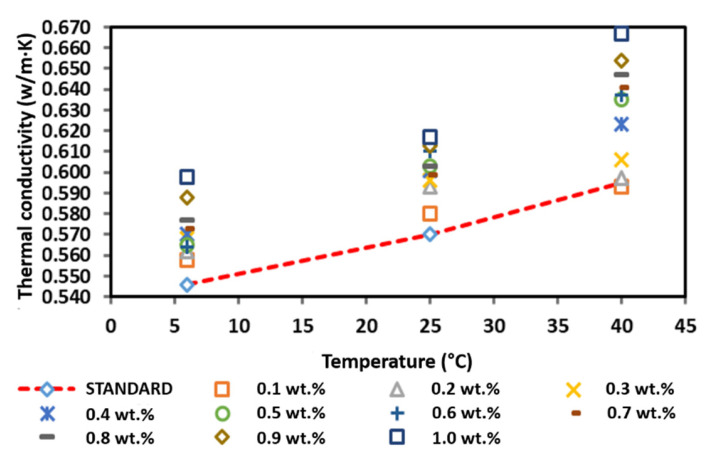
Comparison of the thermal conductivity between the commercial CNF-based nanofluids without PVP and the pure base fluid at various concentrations and temperatures.

Figure 14 and Table 6 present the thermal conductivity of the commercial CNF-based nanofluids without PVP at 6, 25, and 40 °C. Generally, the increased thermal conductivity of the CNF-based nanofluid corresponds with the increasing temperature and weight concentration. Based on Figure 14, the pure base fluid recorded a thermal conductivity of 0.546, 0.570, and 0.595 W/m·K at 6, 25, and 40 °C, respectively. In comparison, the thermal conductivity of the commercial CNF-based nanofluids was higher compared to that of the pure base fluid with the highest thermal conductivity of 0.598, 0.617, and 0.667 W/m·K at 6, 25, and 40 °C, respectively, was achieved at a CNF concentration of 1.0 wt.%. However, the CNF concentration of 0.1 wt.% recorded the only decrement of the thermal conductivity as the temperature increased to 40 °C. A recent study also reported a similar occurrence, which indicated that the increasing thermal conductivity was due to the increasing the nanoparticle volume fraction up 0.6%, and the relation found is usually linear with temperature increased up to 52 °C [31]. This is because the thermal conductivity increasing due to the rising temperature can be considered by Brownian motion and the increase in interactions between the nanoparticles. Additionally, it was clearly found that the thermal conductivity enhancement with volume fraction in the lower volume fraction is more tangible. In fact, at lower concentrations (0.02 to 0.1%), the increase in concentration increases the interactions between nanoparticles. By growing the solid volume fraction in this range, the number of nanoparticles is enhanced, which leads to a rise in surface-to-volume ratio and collisions between nanoparticles. However, nanoparticles may be clustered at higher concentrations (0.1 to 0.6%) by growing the solid volume fraction. The clustering of nanoparticles by adherence forms a thin layer of fluid around nanoparticles and enhances their thermal conductivity. This phenomenon leads to reducing the ratio of surface to volume. As a result, at high solid volume fractions, two factors affect thermal conductivity. These two factors are including the presence of nanotubes as a positive factor (due to its high thermal conductivity), and clustering of nanotubes as a negative factor [32].

### 3.7. Effect of PVP on the Thermal Conductivity of the Surface-Oxidised CNF-Based Nanofluids

**Figure 15:**
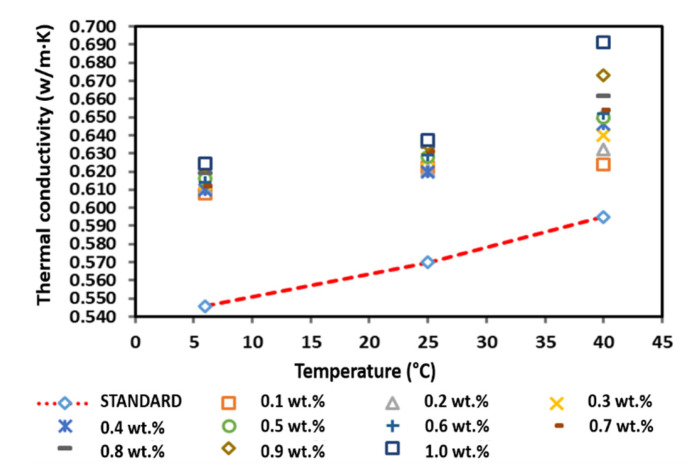
Comparison of the thermal conductivity between the surface-oxidised CNF-based nanofluid without PVP and the pure base fluid at various concentrations and temperatures.

Figure 15 shows the thermal conductivity of the surface-oxidised CNF-based nanofluids without PVP addition. The thermal conductivity of the surface-oxidised CNF-based nanofluids without PVP increased linearly with the increasing temperature owing to the Brownian motion of nanoparticles and was comparatively higher than that of the pure base fluid. According to Figure 15, the thermal conductivity measurement at 6 °C and 25 °C fluctuated with the increasing weight concentration, while the thermal conductivity at 40 °C substantially increased as the weight concentration of the nanoparticles increased. The highest thermal conductivity of 0.624, 0.638, and 0.691 W/m·K at 6, 25, and 40 °C, respectively, was achieved at a CNF concentration of 1.0 wt.%. Conversely, the lowest enhanced thermal conductivity of 0.608 and 0.624 W/m·K was recorded at a CNF concentration of 0.1 wt.% at 6 and 40 °C, respectively. In addition, the lowest thermal conductivity of 0.620 W/m·K at 25 °C was achieved at a CNF concentration of 0.4 wt.%. In short, the positive enhancement achieved in this study was 4.87–16.13%.

Furthermore, Table 7 shows that the highest enhancement percentage of the thermal conductivity of 14.34, 11.84, and 16.13% at 6, 25, and 40 °C, respectively, was obtained at a CNF concentration of 1.0 wt.%. Interestingly, the thermal conductivity pattern of the nanofluid differed from other nanofluids. In particular, the enhancement percentage of the thermal conductivity at 6 and 25 °C did not conform to the increased weight concentration, while the enhancement percentage was linear with the weight concentration at 40 °C. The varying temperature influenced the agglomeration and Brownian motion of nanoparticles, which consequently led to the drastic alteration of the thermal conductivity of the nanofluids. Based on the kinetic theory of particles, the increase in temperature intensifies the Brownian motion, which also increases the kinetic energy of the nanofluid. As a result, the temperature increment promotes the physical interactions between water molecules and nanoparticles. Therefore, the enhanced thermal conductivity would improve the heat transfer process. Figure 15 illustrates the thermal conductivity of the surface-oxidised CNF-based nanofluids without PVP compared to that of the pure base fluid.

**Figure 16:**
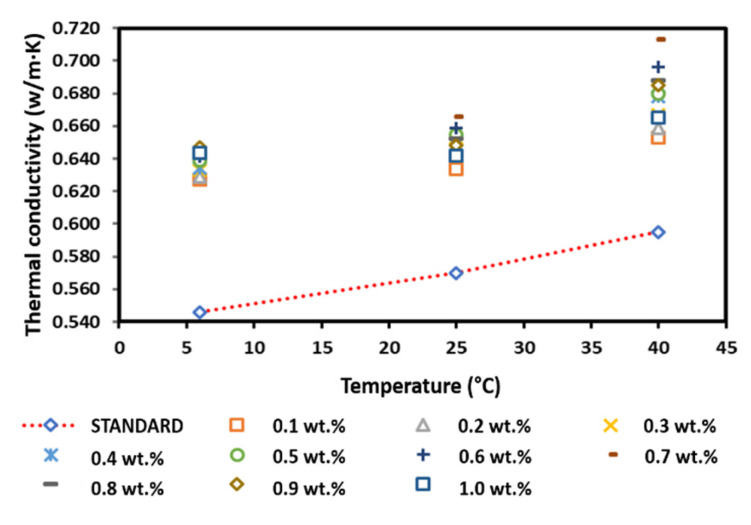
Comparison of the thermal conductivity between the surface-oxidised CNF-based nanofluids with PVP and the pure base fluid at various concentrations and temperatures.

Figure 16 presents the thermal conductivity of the surface-oxidised CNF-based nanofluids with PVP. Although the results showed fluctuations for all temperatures, most of the nanofluid samples exhibited a greater thermal conductivity than that of the pure base fluid. In addition, the increased thermal conductivity varied with the increase in weight percentage of the CNF. It was also noted that the thermal conductivity began to decrease at a CNF concentration of 0.8 and 1.0 wt.% at 25 and 40 °C, respectively, while the thermal conductivity started to decrease at a CNF concentration of 1.0 wt.% at 6 °C. The reducing thermal conductivity was associated with the increasing weight concentration, which increased the viscosity of the nanofluid and potentially influenced the Brownian motion of the suspended nanoparticles [33,34]. Moreover, side effects of PVP addition could also contribute to the decreasing trend of thermal conductivity [35,36]. Nevertheless, the fluctuating thermal conductivity was consistent with the thermal conductivity of the water-based CNT nanofluids as the volume fraction increased [36]. The highest thermal conductivity for all temperatures was recorded at 0.7 and 0.9 wt.%. The highest thermal conductivity at 0.9 wt.% was 0.647 W/m·K at 6 °C, while the highest thermal conductivity at 0.7 wt.% was 0.666 and 0.713 W/m·K at 25 and 40 °C, respectively. Overall, the measured positive enhancement of the surface-oxidised CNF-based nanofluid with PVP addition was achieved at a CNF concentration of 0.1–1.0 wt.%.

**Figure 17:**
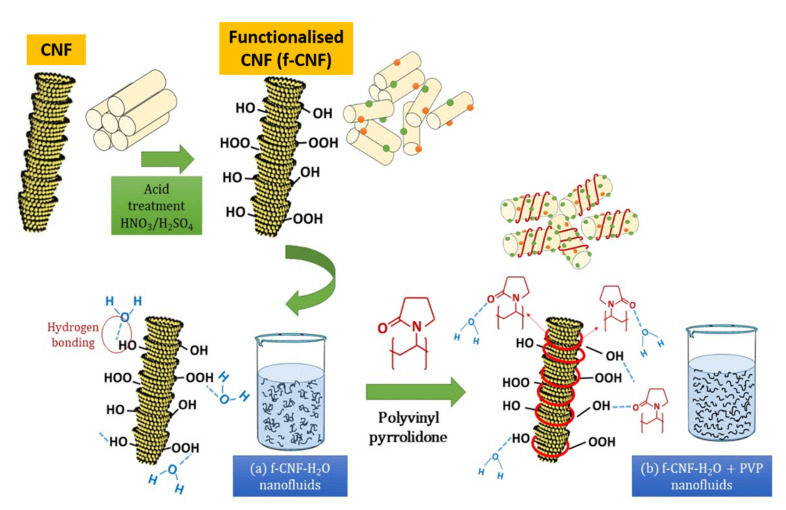
Schematic representation of the surface oxidation and PVP mechanism on the dispersion of CNFs in water-based liquids.

Under normal conditions, the weight concentration increment induces a high thermal conductivity. In contrast, this study showed that the increasing weight concentration did not correlate with the increase in thermal conductivity. According to Table 8, the performance of the surface-oxidised CNF-based nanofluid with PVP recorded an enhancement of 18.50% at 6 °C, while the performance enhancement was 16.84 and 19.83% at 25 and 40 °C, respectively, Interestingly, all three highest enhancement percentages were achieved at a CNF concentration of 0.7 wt.%, indicating the optimum CNF concentration to achieve the highest improvement of the surface-oxidised CNF-based nanofluid with PVP. Hence, the surface oxidation of CNF via the acid treatment significantly enhanced the thermal performance and stability of the nanofluids through the introduction of oxygen functional groups (particularly hydroxyl group and carboxyl group). The surface oxygen functional group was able to stimulate the interaction between water molecules and carbon particles, consequently improving the hydrophilicity of CNF and preventing their sedimentation in ultrapure water. Table 9 presents the summary of comparison for thermal conductivity studies for CNF/water with and without surfactant, cooling liquids and selected nanomaterials in fluids. From the summary in Table 9, it shows that the CNF has high potential use as a nanomaterial in cooling fluids. This is due to the thermal conductivity results that shows a comparable result with other carbonaceous and highly stable metal oxide. Figure 17 illustrates the schematic representation of the surface oxidation and PVP mechanism on the dispersion of CNFs in water-based liquids.

Hence, the surface oxidation of CNF by acid treatment significantly improved the thermal performance and stability of the nanofluids by introducing oxygen functional groups (in particular, the hydroxyl group and the carboxyl group). As illustrated in Figure 17, the surface oxygen functional group was able to stimulate the interaction between water molecules (through hydrogen bonding) and carbon particles, consequently improving the hydrophilicity of CNF and preventing their sedimentation in ultrapure water (Figure 17). The formation of larger aggregates or clusters can lead to sedimentation and a decrease in the thermal conductivity of the nanofluid. Therefore, it is essential to control the aggregation rate so that the stability remains unchanged for a long time by adding stabiliser. The addition of surfactants aims to modify the surface properties of the suspended particles and suppress the formation of clusters of particles to obtain stable suspensions. Therefore, in this work, the addition of PVP surfactant with functionalised CNFs in water-based liquids further improves the thermal stability of the suspensions. This is due to the pyrrolidone group of PVP, which allows for homogeneous incorporation with water molecules by reducing the boundary layer of suspended nanoparticles, preventing them from aggregating over long periods of time, thereby improving energy transfer. (Figure 17).

## 4. Cost Factor Consideration

The beneficial properties of nanofluids lead to high performance in a variety of applications. However, there are also costs associated with the synthesis of nanofluids. Their expensive cost prevents them from being widely used. Nanofluids are measured by the costs associated with their production, such as the costs associated with their preparation and use, and the costs associated with running the instrument while using nanofluids. The reduction in thermal properties due to instability can affect the cost-performance factor of nanofluids. Industrial use of nanofluids is possible only when the cost of nanofluids is lower than the cost savings due to improved performance, i.e., when the thermal conductivity coefficient is moderately high for a long time. Maintaining high thermal conductivity ratio after multiple heating and cooling cycles is important for heat transfer applications. The price–performance factor (PPF) of nanofluids is determined by the thermal conductivity of the nanofluids compared to the base fluids and the price of the nanofluids. Alirezaie et al. [43] reported that the cost of CNTs is much higher than that of metal oxide and hybrid nanofluids. However, ensuring good nanofluid stability over long periods of time is essential to maintain high heat transfer coefficients and PPF over the lifetime of the heating system.

## 5. Conclusions

In conclusion, the structural, morphological, and thermal properties of the surface-oxidised CNF-based nanofluids were successfully improved through the introduction of oxygen functional groups through the surface oxidation treatment and the addition of PVP as the surfactant. The presence of surface oxygen functional groups on the CNF wall was proven based on the higher ID/IG ratio from the Raman spectroscopy. In addition, the TGA curve showed that the additional weight loss was due to the removal of the oxygen functional groups, while the FTIR spectroscopy detected the major bands, such as -OH and C=O, which verified the attachment of oxygenated functional groups on the CNF surface. Based on the overall findings, Method B was considered the best method to synthesise surface-oxidised CNFs and was applied for the nanofluid synthesis. As expected, the commercial CNF-based nanofluids with PVP showed minimal particle sedimentation at a CNF concentration of 0.1 wt.%, which was better than that of commercial CNF-based nanofluids without surfactant, which recorded visible sedimentation at various CNF concentrations. However, the stability of the surface-oxidised CNF-based nanofluid significantly improved. Compared with commercial CNF-based nanofluids without PVP and surface-oxidised CNF-based nanofluid without PVP, the highest enhancement percentage of thermal conductivity was observed at 0.7 wt.% of surface-oxidised CNF-based nanofluid with PVP as significantly improved with the highest enhancement percentage of 18.50, 16.84, and 19.83% at 6, 25, and 40 °C, respectively. Most importantly, the enhanced thermal conductivity of surface-oxidised CNF-based nanofluid with the addition of PVP was contributed by the enhanced stability and homogenised CNF nanoparticles in aqueous media, the increasing temperature from cold condition at 6 to 40 °C, the increasing wt.% of surfactant and the increasing amount of CNF from 0.1 to 1.0 wt.%.

For the factors influenced the thermal conductivity of the nanofluids; (1) it is found that despite very low concentrations of nanoparticles, the thermal properties especially thermal conductivity can be detected. This increase in thermal conductivity could be caused by an increase in Brownian motion of the nanoparticles at higher concentrations. The increase in thermal conductivity is thought to be caused by an increase in Brownian motion, the formation of a nanolayer on the particles, and convection from the particle motion. (2) Increase of temperature increased the thermal conductivity is due to Brownian motion of the nanoparticles in the base fluid. By raising the temperature, the amount of nanofluid kinetic energy will increase thus increasing the physical interaction between the nanoparticle with base media molecules. Thus, this leads to allowing the particles to transfer energy throughout the fluid. Moreover, the increase in thermal conductivity with increase in temperature is caused by an increase of nanoparticle velocity, particle collision, and consequently Brownian motion at higher temperatures.

(3) Surfactants are used to form more stable nanofluids and prevent the nanoparticles from agglomeration and deposition. The initial increase in thermal conductivity with the addition of surfactant can be caused by the fact that nanoparticles become less clustered and can move more freely in the base liquid. An additional layer of surfactant may deposit on nanoparticles by adding further surfactant into base liquid, which prevent heat transfer and energy transfer between nanoparticles hence addition of surfactant can increase thermal conductivity of the nanofluids. These promising characteristics of CNF in water-based fluids would make them accessible as an effective heat transfer media in heating and cooling applications, which are crucial to prevent overheating and permanent damage to electronic or automobile systems.

## Figures and Tables

**Table 1:** Physical and chemical properties of CNF-HHT24.

Properties	Unit	Description
Fibre diameter	nm	100
Iron content	ppm	<100
Polycyclic aromatic hydrocarbons (PAH content)	mg PAH/g fibre	<1
Density	g/cm^3^	2.0
Specific surface area	m^2^/g	41

**Table 2:** Physical and thermal properties of H_2_SO_4_ and HNO_3_.

Properties	Unit	Description	
		H_2_SO_4_	HNO_3_
pH	-	0.3	<1
Density	g/cm^3^	1.84	1.37
Melting point	°C	−20	22

**Table 3:** Physical properties of PVP as the surfactant.

	Unit	Description
Chemical formula	-	(C_6_H_9_NO)_n_
Physical state	-	Crystal powder
Colour	-	White to off-white
Density	g/cm^3^	1.6

**Table 4:** The I_D_/I_G_ ratio of the commercial CNF and surface-oxidised CNF samples.

Sample	I_D_/I_G_ Ratio
Commercial CNF	0.74
CNF-MA	0.81
CNF-MB	0.88
CNF-MC	1.06

**Table 5:** EDX chemical composition of representative CNFs samples.

Sample	Elemental Composition (wt.%)			
	C	O	Al	S
Commercial-CNF	92.42	7.44	0.15	-
CNF-MA	92.52	8.29	0.19	-
CNF-MB	92.95	9.69	0.17	0.16
CNF-MC	79.91	19.52	0.11	0.44

**Table 6:** Thermal conductivity enhancement of the commercial CNF-based nanofluids without PVP.

Weight Concentration of Commercial CNFs (wt.%)	Percentage of Enhancement (%)		
	6 °C	25 °C	40 °C
0.1	2.20 (0.005)	1.75 (0.005)	-
0.2	2.93 (0.002)	4.04 (0.004)	0.3 (0.005)
0.3	4.03 (0.003)	4.56 (0.005)	1.8 (0.004)
0.4	4.40 (0.005)	5.44 (0.005)	4.7 (0.005)
0.5	3.48 (0.006)	5.79 (0.004)	6.7 (0.003)
0.6	3.30 (0.007)	7.02 (0.006)	7.1 (0.005)
0.7	4.95 (0.008)	5.09 (0.005)	7.7 (0.01)
0.8	5.68 (0.005)	5.79 (0.006)	8.7 (0.006)
0.9	7.69 (0.012)	7.54 (0.009)	9.9 (0.01)
1.0	9.52 (0.015)	8.25 (0.01)	12.1 (0.01)

**Table 7:** Thermal conductivity enhancement of the surface-oxidised CNF-based nanofluids without PVP.

Weight Concentration of Surface-Oxidised CNFs (wt.%)	Percentage of Enhancement (%)		
	6 °C	25 °C	40 °C
0.1	11.36 (0.002)	9.16 (0.003)	4.87 (0.001)
0.2	11.90 (0.030)	8.95 (0.003)	6.22 (0.003)
0.3	12.33 (0.005)	10.00 (0.005)	7.56 (0.004)
0.4	11.72 (0.003)	8.77 (0.003)	8.57 (0.005)
0.5	12.82 (0.004)	10.18 (0.006)	9.16 (0.005)
0.6	12.45 (0.005)	10.35 (0.007)	9.58 (0.003)
0.7	12.09 (0.007)	10.70 (0.006)	9.92 (0.004)
0.8	13.37 (0.006)	11.14 (0.009)	11.22 (0.006)
0.9	14.19 (0.008)	11.33 (0.008)	13.11 (0.010)
1.0	14.34 (0.01)	11.84 (0.009)	16.13 (0.01)

**Table 8:** Thermal conductivity enhancement of the surface-oxidised CNF-based nanofluids with PVP.

Weight Concentration of Surface-Oxidised CNFs (wt.%)	Weight Concentration of PVP (wt.%)	Percentage of Enhancement (%)		
		6 °C	25 °C	40 °C
0.1	0.01	14.84 (0.001)	11.19 (0.002)	9.75 (0.01)
0.2	0.02	15.20 (0.001)	12.54 (0.003)	10.76 (0.002)
0.3	0.03	15.93 (0.003)	13.46 (0.002)	12.10 (0.003)
0.4	0.04	16.12 (0.003)	13.77 (0.003)	13.92 (0.003)
0.5	0.05	17.03 (0.003)	14.82 (0.006)	14.29 (0.004)
0.6	0.06	17.44 (0.005)	15.53 (0.005)	16.97 (0.005)
0.7	0.07	17.58 (0.005)	16.84 (0.006)	19.83 (0.006)
0.8	0.08	18.13 (0.006)	14.47 (0.007)	15.63 (0.006)
0.9	0.09	18.50 (0.008)	13.68 (0.006)	15.16 (0.007)
1.0	0.10	17.86 (0.008)	12.63 (0.008)	11.76 (0.007)

**Table 9:** Summary of comparison for thermal conductivity studies for CNF/water with and without surfactant, cooling liquids, and selected nanomaterials in fluids.

Sample	Thermal Conductivity (W/m·K)			Remark		Ref.
	6 °C	25 °C	40 °C	Nanofluid Condition	Stability and Surface Area	
Surface-oxidised CNF/ultra-pure water	0.644 (0.005)	0.666 (0.005)	0.713 (0.006)	0.7 wt.% of CNF, with 10wt.% of surfactant	No sedimentation and agglomeration up to 100 h after addition of PVP as surfactant	This study
Surface-oxidised CNF/ultra-pure water	0.624 (0.01)	0.638 (0.009)	0.691 (0.01)	1.0 wt.% of CNF without surfactant		
CNF/ultra-pure water	0.598 (0.015)	0.667 (0.01)	0.617 (0.01)	1.0 wt.% of CNF without surfactant and without surface treatment		
CNF/Deionised water:Ethylene glycol	0.626	0.635	0.642	−0.5 wt.% of CNF in base fluid composed of DI and EG (90:10), with 10% surfactant	-	[37]
Deionised water	0.546	0.570	0.595	-		
Standard deionised water/Ethylene glycol	0.544	0.551	0.566	-		
Ethylene glycol	0.219	0.223	0.235	-	- Zeta potential values of f-CNT nanofluids possess the highest value of 43.5 mV than the Cu/CNT and Ag/CNT nanofluids. - Cu/CNT and Ag/CNT hybrid nanofluids have been proven to have an excellent stability up to 6 months.	
CNT/Cu/Water	-	0.65 (25 °C)	0.80 (45 °C)	0.05 wt.% of CNT, without surfactant		[38]
CNT/Ag/Water	-	0.67 (25 °C)	0.81 (45 °C)			
CNT/Cu/ Ethylene glycol	-	0.270 (25 °C)	0.285 (45 °C)			
CNT/Ag/ Ethylene glycol	-	0.272 (25 °C)	0.288 (45 °C)			
MWCNT/ ultra-pure water	0.587	0.593	0.626	0.7 wt.% of MWCNT, without surfactant	- The result of stability of commercial MWCNTs based nanofluid with PVP is better compared to without PVP. - Addition of PVP improved the stability of nanofluids and prevented their settlement in water without undergoing acid treatment. - After acid treatment, the surface oxidised MWCNT-based nanofluid with PVP showed better stability.	[39]
MWCNT/ ultra-pure water	0.608	0.628	0.650	0.9 wt.% of MWCNT, with surfactant		
Surface-oxidised MWCNT/ ultra-pure water	0.619	0.640	0.657	0.7 wt.% of MWCNT, without surfactant		
Surface-oxidised MWCNT/ultra-pure water	0.647	0.675	0.693	0.5 wt.% of MWCNT, with surfactant		
SiO_2_/water		0.620	0.72 (50 °C)	1.0 vol.% of SiO_2_ and for hybrid is 70% SiO_2_ and graphene is 30%	- Zeta potential for hybrid nanofluids is 45 mV. - The presence of an isoelectric point in low-pH (3 and 6) values is more likely caused low stability. When pH is higher values (9 and 12), repulsive forces among dispersed nanoparticles are increased and the stability is improved. - Addition of carboxyl methyl cellulose (CMC) as surfactnt imporved the stability and thermal conductivity.	
Graphene/SiO_2_/water		1.24	1.36			[40]
Graphene/water	-	0.740	-	0.5%, without surfactant	- Measured zeta potential of the nanofluid with volume fraction of 0.005% was −47.6 mV which shows high stability of the nanofluid. - Addition of TiO_2_ in graphene/water nanofulids had improved the stability of nanofluids	[41]
TiO_2_/graphene/water	-	0.720	0.790 (45 °C)			
TiO_2_	-	0.700	-			
Al_2_O_3_/SiO_2_/water	-	0.700	0.750	0.5% Al_2_O_3_ + 2.5% SiO_2_	-	[42]

## Data Availability

Not applicable.
